# A dimensional approach to psychosis: identifying cognition, depression, and thought disorder factors in a clinical sample

**DOI:** 10.1038/s41537-025-00641-x

**Published:** 2025-07-14

**Authors:** Mikkel Schöttner Sieler, Philippe Golay, Sandra Vieira, Luis Alameda, Philippe Conus, Paul Klauser, Raoul Jenni, Jagruti Patel, Thomas A. W. Bolton, Patric Hagmann

**Affiliations:** 1 Department of Radiology, Lausanne University Hospital and University of Lausanne (CHUV-UNIL), Lausanne, Switzerland; 2 General Psychiatry Service, Department of Psychiatry, Lausanne University Hospital and University of Lausanne (CHUV-UNIL), Lausanne, Switzerland; 3 Community Psychiatry Service, Department of Psychiatry, Lausanne University Hospital and University of Lausanne (CHUV-UNIL), Lausanne, Switzerland; 4 Institute of Psychology, Faculty of Social and Political Science, University of Lausanne, Lausanne, Switzerland; 5 Department of Psychosis Studies, Institute of Psychiatry, Psychology and Neuroscience, King’s College London, London, UK; 6 Centro Investigacion Biomedica en Red de Salud Mental (CIBERSAM); Instituto de Biomedicina de Sevilla (IBIS), Hospital Universitario Virgen del Rocio, Departamento de Psiquiatria, Universidad de Sevilla, Sevilla, Spain; 7 Center for Psychiatric Neuroscience, Department of Psychiatry, Lausanne University Hospital and University of Lausanne (CHUV-UNIL), Lausanne, Switzerland; 8 Service of Child & Adolescent Psychiatry, Department of Psychiatry, Lausanne University Hospital and University of Lausanne (CHUV-UNIL), Lausanne, Switzerland

**Keywords:** Psychosis, Schizophrenia, Human behaviour

## Abstract

Traditional classification systems based on broad nosological categories do not adequately capture the high heterogeneity of mental illness. One possible solution to this is to move to a multi-dimensional model of mental illness, as has been proposed by the Research Domain Criteria and Hierarchical Taxonomy of Psychopathology frameworks. In this study, we explored the dimensional structure of psychotic disorders. We focused on the question whether combining measures of psychosis with cognitive and depression-related measures results in meaningful, clinically relevant, and valid latent dimensions in a sample of early psychosis (*n* = 113) and chronic schizophrenia patients (*n* = 43, total *n* = 156). We used exploratory factor analysis to identify the symptom dimensions in the Lausanne Psychosis data, a multi-modal prospective data set that includes a broad behavioral assessment of patients diagnosed with psychotic disorders. We evaluated the validity of these dimensions by regressing them to several functioning measures. Our analysis revealed three dimensions: *Cognition, Depression/Negative*, and *Thought Disorder*, explaining 49.2% of the variance. They were related to measures of functioning, the *R*² ranging between 0.38 and 0.42. This study advances the development of a multi-dimensional characterization of psychotic disorders by identifying three symptom dimensions with predictive validity in people with psychosis.

## Introduction

Psychiatric disorders, including psychotic disorders, are defined categorically, as nosologies have been defined for somatic disorders. Conditional on the symptoms they present, patients receive one or multiple diagnoses, and are treated accordingly. This tradition is continued by the newest instantiations of the two most widely used classification systems, the Diagnostic Statistical Manual of Mental Disorders (DSM-5)^1^ and the International Classification of Diseases (ICD-11)^2^. However, this categorical approach has been criticized, as there is high symptom heterogeneity within disorders^3^, and high comorbidity^4^. Instead, some have suggested redefining mental illness as multi-dimensional^5,6^. In this approach, a person can be described by multiple continuous dimensions, which range from healthy to diseased. This is in fact recognized by the world health organization which publishes the ICD, and consequently moved to a dimensional model for personality disorders^2^. Dimensional traits have higher test-retest reliability than categorical measures of psychopathology^7^. Additionally, they may be better predictive targets when predicting from neuroimaging data than when using diagnostic categories as the target^8^. Prediction performance when classifying psychosis patients from healthy controls is poor so far, likely due to high heterogeneity between patients^9^.

## Multi-dimensional frameworks

Notably, there already exist two initiatives that aim to implement such a dimensional approach. First, the Research Domain Criteria (RDoC) framework follows a multi-dimensional approach focusing on the biological causes of mental illness^10^. It tackles basic neurobehavioral processes, aiming to understand mental illness from where they are disrupted. Mental illness is assumed to be dimensional; pathology is understood as extreme deviations on these dimensions^11^.

The second initiative is the Hierarchical Taxonomy of Psychopathology (HiTOP), which takes a theory-free approach. Here, a hierarchy of dimensions is defined based on the correlational structure of symptoms, using factor-analytical methods^5^. This data-driven way of deriving a nosology has the potential to circumvent the reification of expert-derived systems, which rely more strongly on previous knowledge and may thus amplify biases^12^.

## Dimensions of psychosis

Our understanding of primary psychotic disorders could benefit from a multi-dimensional approach. They are characterized by the presence of delusions (fixed false beliefs) and hallucinations (sensory perceptions in the absence of a stimulus), which are not secondary to a somatic disease or the psychological effects of a substance^13^. In the DSM-5, they are summarized as schizophrenia spectrum and other psychotic disorders. Defining these disorders as part of a spectrum is a first step towards a multi-dimensional view, corroborated by the inclusion of a Psychosis Symptom Severity Scale, where the occurrences of different symptoms are rated on a five-point scale^1^. Still, the spectrum consists of several discrete diagnoses that differ in severity and symptom profile. Test-retest reliability was high for a dimensional measure of psychosis^14^, but lower for schizophrenia, schizoaffective disorder, and bipolar I disorder as per the DSM-5^15^, which can be seen as evidence in favor of a multi-dimensional approach. Internal consistency was also higher for dimensional psychosis traits when directly compared to DSM diagnoses^16^. Evidence also suggests that the psychosis dimension continues into the healthy spectrum. A study investigating how well personality traits align with schizophrenia symptoms and schizotypal traits found that the model combining them provided the best fit^17^. This notion is also supported by the prevalence of transient psychotic experiences, especially during adolescence, that do not develop into a psychotic disorder, which has been taken as evidence of a psychosis continuum^18^.

In the HiTOP framework, a psychosis superspectrum is hypothesized, which comprises two spectra: thought disorder (psychoticism) and detachment^19^. Each spectrum, in turn, consists of a number of maladaptive traits, which are stable over time, and symptoms, which can be more transient. They were found by aggregating structural (i.e., factor-analytical) evidence from several studies.

While this structure presents one way to delineate the different dimensions that characterize psychotic disorders, it is still work-in-progress. It should be tested on different samples and with different measures, as it should ideally be independent of the exact instruments or indicators used. This is in line with two of the goals of RDoC^11^: to refine the phenotypes of psychotic disorders (pillar two) and to find reliable and valid measures (pillar three). The reliability and validity of not only the measurement instruments, but also higher-level summary dimensions, should be investigated. In other words, the meta-properties of these measures, how they relate to each other, and what underlying constructs might influence each of them, are all of interest.

This study has the goal to investigate this meta-structure of symptoms in psychotic disorders in a sample of early psychosis and chronic schizophrenia patients. Much previous research has focused on the latent structure of psychosis symptoms as measured using the Positive and Negative Symptom Scale (PANSS), where a five-factor structure has been established (for a meta-analysis, see Lim et al.^20^). Some of this work made use of samples consisting of early psychosis patients^21,22^, or including them in addition to chronic schizophrenia patients^23^. However, these studies did only include the PANSS items in their model, not other measures of symptoms or cognition. Some studies already investigated the meta-structure of symptoms in early psychosis patients with a wider array of measurements. One study found factors for internalizing, externalizing, and schizophrenic symptoms^24^.

The advantage of multi-dimensional conceptualizations of mental illness is that they allow the characterization of the whole clinical presentation of patients, without having to worry whether they fit into strictly defined classes. Comorbidities are high in schizophrenia, especially for depressive symptoms, which occur in about 50% of patients^25^. Schizophrenia patients show a range of cognitive symptoms^26^, which have shown to be related to psychotic symptoms^27^. The heterogeneity of psychotic disorders means that for these comorbidities, multiple diagnoses need to be given to a patient, even though it might be questionable whether the symptoms occur due to independent causes. In a multi-dimensional framework, this sort of heterogeneity can be modeled natively. Since previous research showed that depression and cognitive impairment are clearly part of the clinical presentation, it might be indicated to include measures of each as part of a multi-dimensional model of primary psychotic disorders.

This study thus seeks to identify symptom dimensions in psychosis patients, their relationship, and whether they relate to real-life functioning. We investigated this in a sample consisting of both early psychosis and chronic schizophrenia patients, covering a broad range of symptom severity and disease progression. To find the latent dimensions in psychosis, we used exploratory factor analysis (EFA), comparing the resulting latent structure to the one proposed in the HiTOP framework. Correlations between factors gave indication of whether these dimensions are part of the same clinical presentation. Finally, the validity of factors was examined by relating the factor scores to real-life functioning measures in patients with early phase psychosis and schizophrenia.

## Materials and methods

### Subjects

This study considers 226 patients from the Lausanne Psychosis Cohort, of whom 169 were early psychosis patients (i.e., within 5 years after a first psychotic episode) recruited from the Treatment and Early Intervention in Psychosis Program (TIPP) in Lausanne, Switzerland^28^. Inclusion criteria for recruitment into TIPP were to be between 18 and 35 years old, live in the catchment area of the Lausanne University Hospital (Centre Hospitalier Universitaire Vaudois, CHUV), and meet the psychosis criteria from the Comprehensive Assessment of At-Risk Mental States (CAARMS) instrument, defined by the “psychosis threshold” subscale^29^. Additionally, 63 chronic schizophrenia patients meeting the DSM-IV criteria for schizophrenia or schizoaffective disorder were recruited from the CHUV. The data collection for TIPP was approved by the Human Research Ethics Committee of the Canton of Vaud. Data collection agreement for the schizophrenia patients was given by the local ethical committee. Informed consent was given by all participants in the study.

Of the 226 patients, 169 (75%) were male and 57 (25%) female, with a mean age of 28 (SD = 8.5). Due to the naturalistic nature of the cohort, many patients had missing data. We employed a two-step strategy to mitigate this. First, we excluded *patients* with more than 50% of values from the different measurement instruments missing. Second, we excluded *variables* with more than 25% of patients missing. This way, no variable had more than 25% missing values, while retaining as many subjects as possible for further analysis. The rest of the missing data was estimated using an imputation technique (see section “Preprocessing”).

The final sample consisted of 156 subjects, 113 (72.4%) of which were early psychosis patients, and 43 (25.6%) of which were schizophrenia patients. The mean age during the clinical evaluation was 24.4 (SD = 5.3). Of the patients, 120 (76.9%) were male and 36 (23.1%) female. The characteristics of each cohort and the combined sample can be seen in Table 1.

**Table 1:** Demographic and clinical characteristics for the early psychosis cohort, schizophrenia cohort, and combined sample.

		Early Psychosis (*N* = 113)	Schizophrenia (*N* = 43)	Combined Sample (*N* = 156)
Gender (%)	M F	87 (80%) 26 (20%)	33 (80%) 10(20%)	120 (80%) 36 (20%)
Age		24.4 (5.3)	36.5 (9.1)	27.7 (8.5)
PANSS Wallwork Positive		6.8 (3.0)	8.2 (3.5)	7.2 (3.2)
PANSS Wallwork Negative		15.3 (5.7)	14.5 (5.3)	15.1 (5.6)
PANSS Wallwork Disorganized		5.7 (2.2)	6.7 (2.2)	5.9 (2.2)
PANSS Wallwork Excited		5.9 (2.2)	6.6 (2.5)	6.1 (2.3)
PANSS Wallwork Depressed		8.3 (2.9)	8.1 (2.9)	8.3 (2.9)
MADRS Depression		12.9 (8.4)	14.2 (8.8)	13.3 (8.5)
YMRS Mania		2.7 (3.7)	3.8 (3.8)	3.0 (3.7)
Rosenberg Self-Esteem		27.8 (6.5)	27.6 (6.1)	27.7 (6.4)
MATRICS Processing Speed		38.9 (13.2)	35.0 (11.9)	37.8 (12.9)
MATRICS Sustained Attention		41.2 (10.9)	38.6 (10.6)	40.8 (10.8)
MATRICS Working Memory		46.2 (9.7)	42.8 (9.9)	45.3 (9.9)
MATRICS Verbal Learning		44.0 (10.1)	42.1 (8.8)	43.5 (9.7)
MATRICS Visual Learning		47.5 (10.7)	41.9 (13.9)	46.0 (11.9)
MATRICS Problem Solving		46.9 (11.0)	46.9 (11.8)	46.9 (11.2)
Clinical Global Impression		3.7 (0.9)	3.9 (1.0)	3.7 (0.9)
Global Assessment of Functioning		55.3 (11.7)	54.5 (11.8)	55.1 (11.7)
Social and Occupational Functioning		56.7 (12.2)	55.0 (11.1)	56.2 (11.9)

Mean (standard deviation) for all measures except gender, which is shown as count (percentage) for male and female.

*M* male, *F* female, *PANSS* Positive and Negative Symptoms Scale, *MADRS* Montgomery–Åsberg depression rating scale, *MATRICS* Measurement and Treatment Research to Improve Cognition in Schizophrenia, *YMRS* Young Mania Rating Scale, *CMRS* Case Manager Rating Scale.

### Instruments

#### Baseline instruments used for EFA

A total of 16 measurement instruments were used in the factor analysis. One composite score per instrument was entered as a variable in the EFA. The only exception was the PANSS, where we used the factor scores by Wallwork and colleagues^30^, which have been shown to be stable over time^31^. It consists of five factors: *Positive*, *Negative*, *Disorganized*, *Excited*, and *Depressed*. Furthermore, the Montgomery–Åsberg depression rating scale (MADRS; Montgomery & Åsberg, 1979)^32^, the Young Mania Rating Scale (YMRS; Favre et al.^33^), and the Rosenberg self-esteem scale^34^ were used.

We also included six scores from the Measurement and Treatment Research to Improve Cognition in Schizophrenia (MATRICS) Consensus Cognitive Battery (MCCB)^35,36^, corresponding to processing speed, working memory, verbal learning, sustained attention, problem solving, and visual learning.

Additionally, three measures were kept separate to investigate how the factors relate to real-life functioning. These measures were the Clinical Global Impression (CGI) scale^37^, the Global Assessment of Functioning (GAF) scale^38^, and the Social and Occupational Functioning Assessment Scale (SOFAS) from the DSM-IV^39^.

### Preprocessing

The missing values were imputed using the Multivariate Imputation by Chained Equations algorithm^40^, which iteratively predicts the missing values in one column from the remaining columns using a linear regression model. Age and gender were regressed out from each variable and the residuals were used for further analysis. This was in order to find factors that are independent of age and gender of participants. The factor analysis was additionally run with variables where this step was not performed. Variables were z-scored.

### Factor analysis

The latent structure of the preprocessed variables was modeled via EFA with principal axis factoring and promax rotation^41^. The analysis was carried out using JASP^42^. Loadings were interpreted according to the guidelines in Cohen, 1992^43^, with values ≥ 0.1 and < 0.3 indicating a small effect, values ≥ 0.3 and < 0.5 a medium effect, and values ≥ 0.5 a large effect.

We used parallel analysis^44^ to find the optimal number of factors. This method compares the eigenvalues of factor solutions with different numbers of factors to those from simulated data. The point where the eigenvalues of the real data become lower than the simulated eigenvalues is chosen as the optimal factor number. Additionally, factors needed to be non-trivial, defined as having loadings > 0.3 for more than two variables. They also should concurrently explain as much variance as possible. Finally, the interpretation and meaning of each factor was also verified. This ensured that the rotation was able to recover a meaningful and simple structure. Since promax is an oblique rotation, we additionally calculated correlations between factors.

#### Hierarchy

We explored the hierarchical structure of factors using hierarchical EFA^45^, which extends EFA beyond the solution deemed optimal by our criteria. In this method, factor solutions for one, two, up until a maximum number of factors are calculated. We chose this number as the number of factors for which the criteria beyond parallel analysis were still fulfilled (non-triviality, interpretability). These different factor solutions are viewed as levels in a hierarchy. For each of those levels, factor scores were extracted, giving an estimate of where each subject falls on each factor. These factor scores were then correlated between consecutive levels, the correlations being interpreted as describing the “flow” of variance when going up in the hierarchy.

We used JASP software^42^ to estimate factor scores for each participant, and calculated the correlations between all levels where factors were non-trivial and interpretable in Python.

### Relation to functioning measures

The association between the best EFA solution and functioning was investigated with a Bayesian linear regression. In contrast to ordinary least squares regression that simply finds the model parameters that give the best fit to the data, in a Bayesian linear regression, for each parameter a probability distribution is estimated. This also allows us to compare the relative plausibility of each model^46^. For each functioning measure, a regression model was fitted with the factor scores as independent variables, implemented in JASP^42^. Model fit was assessed using the coefficient of determination (*R*²), and the Bayes Factor, which quantifies how much more likely a given model is than the null model^47^. Of note, the Bayes Factor indicating evidence *against* a model is the inverse of the Bayes Factor indicating evidence *for* a model, i.e., $B{F}_{01}=\frac{1}{B{F}_{10}}$, meaning that statements can be made both about the presence and absence of an effect. For the estimation, we used a uniform distribution as a prior for the model. This is a flat prior that does not make any assumptions about the resulting distribution.

## Results

### Optimal EFA

**Fig. 1:**
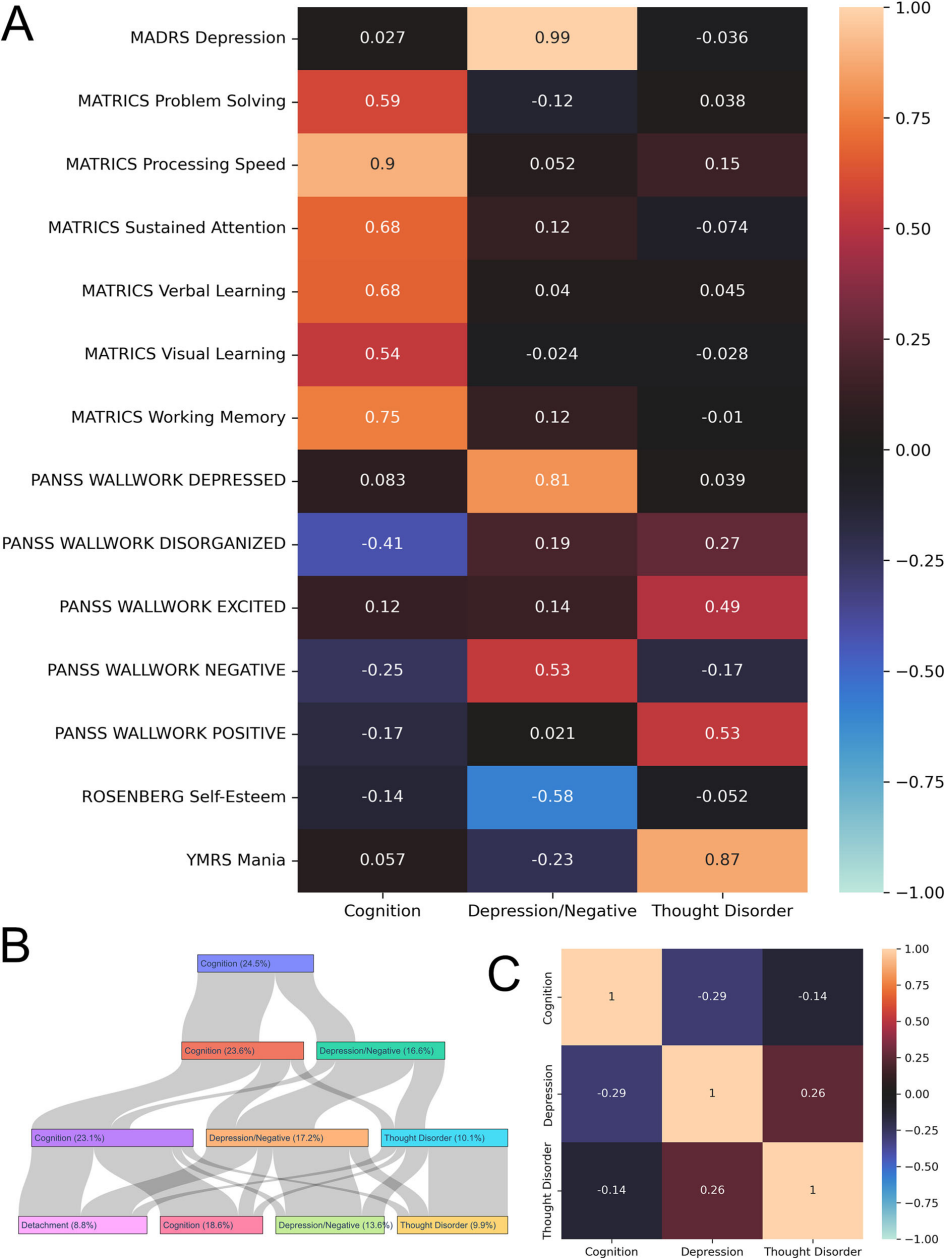
Results of the exploratory factor analysis (EFA). **A** Factor loadings of the four-factor solution. The variables are in the rows and the factors in the columns. Factors were named based on the content of the highest-loading variables. **B** Hierarchy of the factors. Numbers in brackets show the percentage of variance explained by each factor. The width of the bar shows the correlation between factors of consecutive levels in the hierarchy, which can be interpreted as the “flow” of variance. **C** Factor correlations. Pearson correlation coefficients between the factor scores of each factor in the three-factor solution.

According to our criteria, the factor solution with three factors was optimal, according to the parallel analysis as seen in Fig. S1. At the same time, there were at least three variables with loadings >0.3, thus making all factors non-trivial and not under-defined. This model explained 49.2% of the total variance in the data. The factor loadings can be seen in Fig. 1A, and the loadings for the factor solutions with one to four factors, in Tables 1–4 in the Supplementary information. The salient loadings give an indication to the content of each factor.

The first factor had high loadings from all cognitive scores, and a medium negative loading from the PANSS Disorganized variable. We called this factor *Cognition* for the primary loading pattern of cognitive variables. Disorganized symptoms likely influence cognitive performance, and are thus related to cognition, but not the primary content of this factor.

The second factor had high loadings from both the PANSS and the MADRS depression items, as well as a high negative loading from the Rosenberg Self-Esteem questionnaire. We also observed a large loading from the PANSS Negative Symptoms scale. We decided to interpret this factor as *Depression/Negative* due to the very high loadings from the two depression scores and the negative symptom measure.

The third factor had high loadings from the PANSS Positive symptoms scale as well as the YMRS mania scale. It also showed a medium loading value from the PANSS Excited and a small loading from the Disorganized dimension. In line with the results from Kotov and colleagues^19^, we called this factor *Thought Disorder*.

### Hierarchy of factors

While three was deemed to be the optimal number of factors according to our criteria, up to four factors were non-trivial and interpretable. Figure 1B shows the hierarchy of the factors, when going from one factor to four. Following the original work from Goldberg and colleagues^45^, the correlation between factor scores of consecutive levels of the hierarchy can be interpreted as the “flow” of variance when going down the hierarchy and can be seen in Table S5. In this case the factors explained mostly variance that was previously unaccounted for. This can be seen by the inflow in the nodes in the figure, which was very small for almost all the factors that appear new at each level. For example, at level two, the *Depression/Negative* factor had only little inflow from the *Cognition* factor, and thus explained mostly variance not accounted for by the *Cognition* factor of level one. This was similar to the *Thought Disorder* factor that emerged at level three. The only exception to this was the *Detachment* factor, which emerged at level four. As can be seen from the loading matrix for the four-factor solution in Table S4, this factor (factor 3) received a high loading from the PANSS Negative symptoms, as well as medium loadings from PANSS Disorganized symptoms (positive) and MATRICS Visual Learning (negative). It explained variance that was previously explained by *Cognition* and *Depression/Negative*.

### Factor correlations

As seen in Fig. 1C, the strongest correlation was between *Cognition* and *Depression/Negative*, which showed a weak negative correlation. The factor correlations further indicated that *Cognition* and *Thought Disorder* were weakly anti-correlated, and that *Depression/Negative* and *Thought Disorder* were weakly correlated.

### Relation to functioning measures

The results from the regression analysis are summarized in Table 2. The regression models showed decisive evidence that the factor scores were related to the CGI, GAF, and Social and Occupational Functioning (BF_10_ > 100). In all three cases, the model including all three factor scores had the highest R², indicating that they each offer valuable information to explain the variance in the respective functioning measure. Note that for all three of these functioning variables, for at least two of the beta weights of factor scores the 95 percent credible interval did not include zero, indicating that multiple factors are needed to accurately characterize the relationship with functioning measures. In the model for CGI, the credible interval excluded zero for all three factors, while for GAF and SOFA, the beta weights for *Cognition* and *Depression/Negative* were unlikely to be zero.

**Table 2:** Regression Models – Relation to Functioning Measures.

Models	BF_10_	*R*²	Beta Cognition	Beta Depression/Negative	Beta Thought Disorder
Clinical Global Impression	8.7 × 10^14^	0.42	−0.22 [−0.36, −0.08]	0.39 [0.25, 0.51]	0.32 [0.18, 0.44]
Global Assessment of Functioning	6.2 × 10^12^	0.38	0.25 [0.1, 0.39]	−0.47 [−0.6, −0.33]	−0.04 [−0.22, 0]
Social and Occupational Functioning Assessment	1.34 × 10^15^	0.42	0.31 [0.19, 0.45]	−0.49 [−0.6, −0.34]	0.00 [−0.06, 0.03]

*Note*. Summary statistics of the Bayesian regression models. The models are named after the dependent variable. Independent variables for each model are the factor scores for the three factors *Cognition, Depression/Negative*, and *Thought Disorder*. The beta columns represent the beta coefficients for each independent variable averaged over the posterior distribution, with the 95 credible interval in square brackets.

*P* probability, *M* model, *BF* Bayes Factor, *R*^²^ coefficient of determination.

## Discussion

This study investigated the latent factor structure of a broad array of cognitive and symptom measurements in a dataset of early psychosis and schizophrenia patients. We found that three meaningful and interpretable factors presented an optimal solution, with factors related to *Cognition*, *Depression/Negative*, and *Thought Disorder*. When looking at the hierarchical structure of these factors, new factors in each level mostly explained variance previously unaccounted for, except for *Detachment*, which combined variance from *Cognition* and *Depression/Negative* from the previous level. All factors showed weak correlations with each other. When relating the factor scores to functioning measures, we found that there was evidence for a linear relationship with measures of functioning and clinical impression.

The structure of the factor solution and the content of the factors is in line with what has been established in the literature. The *Cognition* factor has clear loadings from all cognitive tests, analogous to the long-established general mental ability^48^ which has been shown to be universal across cultures and languages^49^. *Cognition* is also related to disorganized symptoms. In this sample, disorganized symptoms are measured with the specific symptoms conceptual disorganization (e.g., circumstantiality, loose associations, tangentiality, gross illogicality or thought block), difficulty in abstraction, and poor attention^30^. All of these symptoms will negatively influence cognitive performance.

The second axis of variation in our psychosis sample is on the *Depression/Negative* dimension, with depression-related items on one end of the spectrum, and self-esteem on the other, forming a positive-negative axis. Depression is highly comorbid in psychosis^50^, thus it is not surprising that the *Depression/Negative* factor emerges as one of the central axes. Notably, there is also a loading from the negative symptoms variable. This is expected, given the partial conceptual overlap between depression and negative symptoms. While different from depressive symptoms, they share common aspects such as loss of interest and anhedonia, which might explain the weak association to this factor.

Third, there is the *Thought Disorder* factor, characterized mostly by mania, positive symptoms, excitedness, and disorganized thinking. The study by Kotov and colleagues^19^ raised the question of whether mania constitutes its own dimension or is part of the thought disorder spectrum. Like in their provisional model, our results suggest that mania should be viewed as part of thought disorder, as here mania loads onto the same factor as positive symptoms, excitedness, and disorganized thinking. More research on other samples is therefore needed to bring a definitive answer to this issue.

While largely in agreement with the structure outlined in ref. ^19^, our results differ in important ways. First, we include a dimension for *Cognition*. Cognitive deficits are a hallmark of psychosis^51^, and are an important predictor of functioning^52^. Disorganized symptoms also load highly on this factor, which is unsurprising given the well-established link between cognitive deficits and disorganized thinking^53–57^.

A different case is *Depression/Negative*, which corresponds to the internalizing dimension in the HiTOP framework. However, in their model, it is not directly linked to the psychosis superspectrum^19^, but has been found as one of the central dimensions of variation in a sample of early psychosis patients^24^. The *Depression/Negative* factor is correlated to both *Cognition* and *Thought Disorder*. This is consistent with the fact that major depressive disorder is a major comorbidity in schizophrenia^25,58^.

Overall, the three factors bear some resemblance to the original three-factor model of schizophrenia symptoms by Peter Liddle^59^. Comparing the factors from our study to the ones from this landmark study, one can find analogy between our *Cognition* and their disorganization factor, our *Depression/Negative* and their psychomotor poverty factor, and our *Thought Disorder* and their disintegrative reality distortion factor. Of course, the measurement instruments from this study are partly based on these previous results. Still, our study reproducing factors very similar to these original symptom dimensions might indicate that they constitute a higher level of abstraction than is now commonly used when characterizing the symptom dimensions in psychotic disorders.

That being said, the hierarchy of factors in our study painted a slightly different picture. It revealed a *Detachment* factor, explaining variance that was included in *Cognition* and *Depression/Negative* factors in the previous level. This could be due to negative symptoms, which constitute the main variable in *Detachment*, influence cognition^60^, and overlap with depression as mentioned before. However, since this factor only emerged when going beyond the number of factors given by the parallel analysis, this factor should be interpreted with caution.

Another important result from our study is that models including multiple factor scores as independent variables show the highest model fit of the models characterizing the relationship to functioning measures. This once more underscores the point that a multidimensional approach is needed to fully characterize mental illness and the influence it has on patients’ lives. Some findings are interesting to point out here. Firstly, *Thought Disorder* was only related to clinical impression, but not to the other two functioning measures. While symptom severity has been shown to influence the quality of life of psychosis patients^61^, our results indicate that cognitive impairment and depressive symptoms in particular are strongly related to functioning measures. *Depression/Negative* also had the strongest relationship to all three functioning measures, underscoring its importance in characterizing patients with psychosis. This is corroborated by the literature, which has shown that quality of life is consistently related to depressive symptoms^62^. *Cognition* was also related to all three functioning measures, even though the beta weights were smaller in this case. This is in line with previous research as well, where it has been shown that cognitive measures were related to functioning measures^63,64^.

One limitation to this study is that there was only patient data available for the full range of measures. We thus do not know where patients fall on the proposed dimensions relative to healthy subjects. While there exists a control group for the Lausanne psychosis cohort, not all measurements that are available for the patient data also exist for the controls. Thus, we opted to limit our analysis to the early psychosis and schizophrenia subgroups. It would have been advantageous to have a broader variety of patients with different diagnoses that include symptoms of thought disorder, as is for example the case in bipolar disorder^1^. Of course, the approach taken in this study assumes that subjects differ on the same dimensions to begin with. In other words, we assume that the difference between early psychosis and chronic schizophrenia patients is quantitative, and not qualitative. This can lead to measurement invariance^65^, which we were not able to test for due to the small sample size of the schizophrenia cohort.

The fact that there was only patient data available also points to an avenue for future research. Developing a normative model of these dimensions on a sample that includes the same measurements both for healthy participants and patients, preferably with a broader range of diagnoses, would provide a more general view of the central dimensions in the context of psychosis. This way, one could characterize patients in their deviation from a normal range, which relates to the second RDoC pillar. A second goal could be to investigate how the identified dimensions relate to functioning measures at later points in time, to see if they provide predictive value in terms of prognosis or disease progression. Related to that, one could also make use of the factor scores as *targets* of prediction, using neuroimaging or other biological measures as predictors.

The goal of this paper was to explore the dimensions that characterize symptoms in psychosis. We identified three dimensions that optimally summarized the data, namely *Cognition*, *Depression/Negative*, and *Thought Disorder*. Validity was demonstrated through their relationship to measures of clinical impression and functioning. Our results add to existing dimensional models of psychosis in two important ways. First, *Cognition* and *Depression/Negative* could be used as additional dimensions for characterizing patients with psychosis. While *Thought Disorder* might be considered as the dimension that is unique to psychotic disorders, our results show considerable variability in the other two, and *Depression/Negative* is particularly related to functioning measures. Second, we do not find mania to be a separate dimension, but instead on the same axis as positive and excited symptoms. Future research will shed light on the predictive value of these dimensions, whether they generalize to healthy populations, and how they relate to biological measures.

## Supplementary information

Supplementary information

## Data Availability

Due to ethical restrictions, the data used in the current study are not publicly available. Interested parties may request access from the corresponding author through a reasonable inquiry, subject to approval by the Regional Ethics Committee.

## Code availability

All analysis code can be found in the following repository: https://github.com/mschoettner/The-Multi-Dimensional-Structure-of-Psychotic-Disorders.
