# Supplementary material for: A dimensional approach to psychosis: identifying cognition, depression, and thought disorder factors in a clinical sample
Source: Schizophrenia (Heidelb). 2025 Jul 14;11(1):97. doi: 10.1038/s41537-025-00641-x (PMC12259953; doi:10.1038/s41537-025-00641-x)
Supplement: Supplementary file 1 — Supplementary information [file 41537_2025_641_MOESM1_ESM.docx]

Supplementary Information


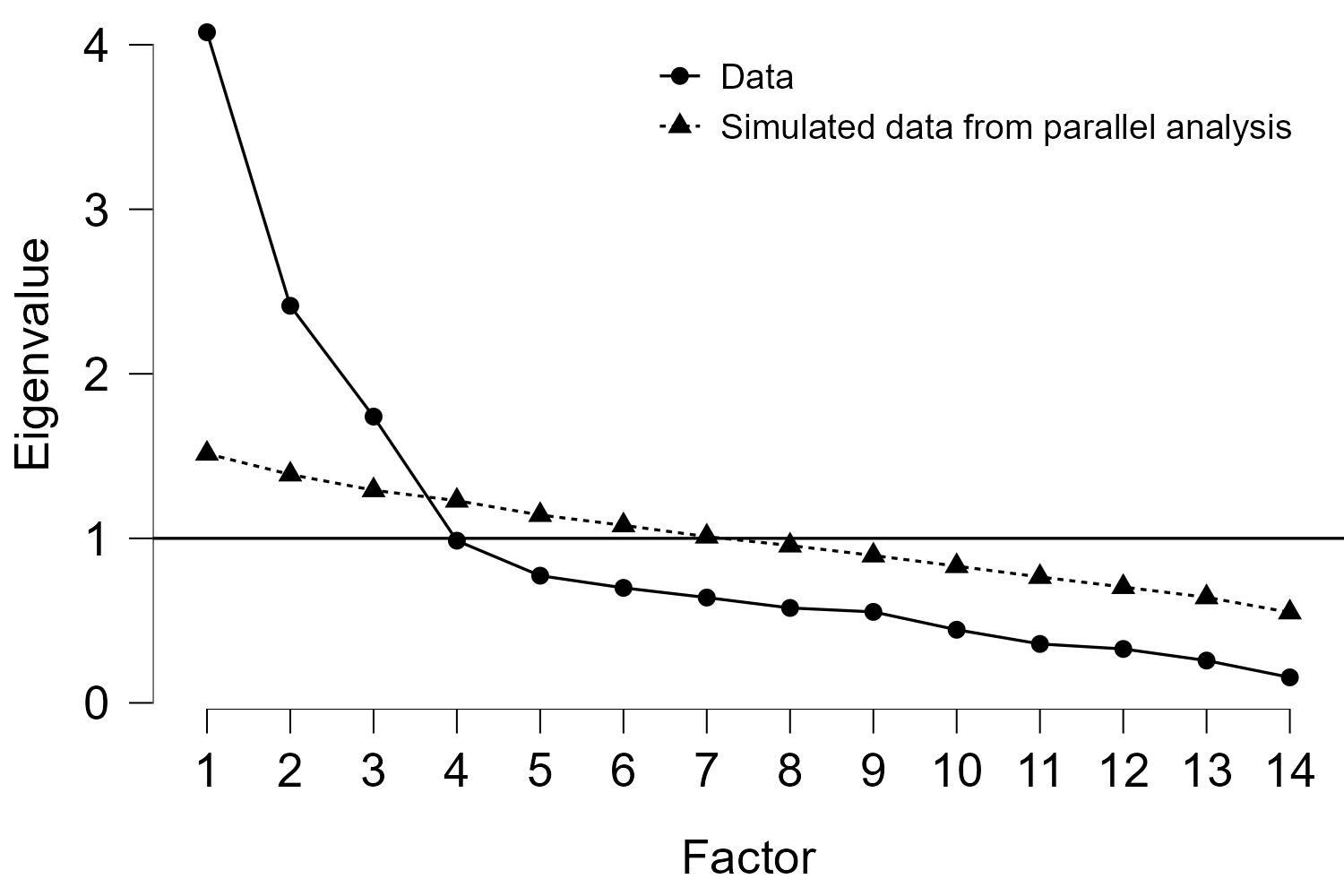


Figure S1: Scree plot showing the results from the parallel analysis. Notice the crossover point between the real data (circles) and the simulated data (triangles) going from three to four factors.

| Table S1: Factor Loadings 1-Factor Solution | | | | | |
| --- | --- | --- | --- | --- | --- |
|  | | Factor 1 | | Uniqueness | |
| MATRICS Processing Speed |  | 0.751 |  | 0.437 |  |
| MATRICS Problem Solving |  | 0.634 |  | 0.598 |  |
| MATRICS Working Memory |  | 0.630 |  | 0.603 |  |
| MATRICS Verbal Learning |  | 0.604 |  | 0.635 |  |
| MATRICS Sustained Attention |  | 0.586 |  | 0.657 |  |
| PANSS WALLWORK DISORGANIZED |  | -0.581 |  | 0.662 |  |
| MATRICS Visual Learning |  | 0.538 |  | 0.710 |  |
| PANSS WALLWORK NEGATIVE |  | -0.494 |  | 0.756 |  |
| MADRS Depression |  | -0.459 |  | 0.790 |  |
| PANSS WALLWORK DEPRESSED |  | -0.361 |  | 0.870 |  |
| PANSS WALLWORK POSITIVE |  | -0.298 |  | 0.911 |  |
| ROSENBERG Self-Esteem |  | 0.212 |  | 0.955 |  |
| PANSS WALLWORK EXCITED |  | -0.083 |  | 0.993 |  |
| YMRS Mania |  | -0.033 |  | 0.999 |  |
|  | | | | | |
| Note. Applied rotation method is promax. | | | | | |

| Table S2: Factor Loadings 2-Factor Solution | | | | | | | |
| --- | --- | --- | --- | --- | --- | --- | --- |
|  | | Factor 1 | | Factor 2 | | Uniqueness | |
| MATRICS Processing Speed |  | 0.893 |  | 0.160 |  | 0.256 |  |
| MATRICS Working Memory |  | 0.754 |  | 0.160 |  | 0.472 |  |
| MATRICS Sustained Attention |  | 0.684 |  | 0.130 |  | 0.564 |  |
| MATRICS Verbal Learning |  | 0.682 |  | 0.098 |  | 0.562 |  |
| MATRICS Problem Solving |  | 0.610 |  | -0.066 |  | 0.601 |  |
| MATRICS Visual Learning |  | 0.548 |  | -0.001 |  | 0.700 |  |
| PANSS WALLWORK DISORGANIZED |  | -0.446 |  | 0.260 |  | 0.670 |  |
| PANSS WALLWORK NEGATIVE |  | -0.287 |  | 0.424 |  | 0.672 |  |
| PANSS WALLWORK POSITIVE |  | -0.196 |  | 0.193 |  | 0.903 |  |
| ROSENBERG Self-Esteem |  | -0.086 |  | -0.596 |  | 0.666 |  |
| PANSS WALLWORK EXCITED |  | 0.079 |  | 0.299 |  | 0.917 |  |
| MADRS Depression |  | -0.064 |  | 0.917 |  | 0.123 |  |
| YMRS Mania |  | 0.022 |  | 0.090 |  | 0.992 |  |
| PANSS WALLWORK DEPRESSED |  | 0.012 |  | 0.811 |  | 0.348 |  |
|  | | | | | | | |
| Note. Applied rotation method is promax. | | | | | | | |

| Table S3: Factor Loadings 3-Factor Solution | | | | | | | | | |
| --- | --- | --- | --- | --- | --- | --- | --- | --- | --- |
|  | | Factor 1 | | Factor 2 | | Factor 3 | | Uniqueness | |
| MATRICS Processing Speed |  | 0.897 |  | 0.052 |  | 0.153 |  | 0.230 |  |
| MATRICS Working Memory |  | 0.750 |  | 0.122 |  | -0.010 |  | 0.474 |  |
| MATRICS Sustained Attention |  | 0.681 |  | 0.123 |  | -0.074 |  | 0.556 |  |
| MATRICS Verbal Learning |  | 0.675 |  | 0.040 |  | 0.045 |  | 0.564 |  |
| MATRICS Problem Solving |  | 0.592 |  | -0.120 |  | 0.038 |  | 0.601 |  |
| MATRICS Visual Learning |  | 0.535 |  | -0.024 |  | -0.028 |  | 0.701 |  |
| PANSS WALLWORK DISORGANIZED |  | -0.415 |  | 0.193 |  | 0.271 |  | 0.612 |  |
| PANSS WALLWORK NEGATIVE |  | -0.248 |  | 0.533 |  | -0.174 |  | 0.606 |  |
| PANSS WALLWORK POSITIVE |  | -0.175 |  | 0.021 |  | 0.527 |  | 0.658 |  |
| ROSENBERG Self-Esteem |  | -0.138 |  | -0.582 |  | -0.052 |  | 0.673 |  |
| PANSS WALLWORK EXCITED |  | 0.125 |  | 0.135 |  | 0.487 |  | 0.722 |  |
| PANSS WALLWORK DEPRESSED |  | 0.083 |  | 0.810 |  | 0.039 |  | 0.360 |  |
| YMRS Mania |  | 0.057 |  | -0.225 |  | 0.868 |  | 0.300 |  |
| MADRS Depression |  | 0.027 |  | 0.989 |  | -0.036 |  | 0.054 |  |
|  | | | | | | | | | |
| Note. Applied rotation method is promax. | | | | | | | | | |

| Table S4: Factor Loadings 4-Factor Solution | | | | | | | | | | | |
| --- | --- | --- | --- | --- | --- | --- | --- | --- | --- | --- | --- |
|  | | Factor 1 | | Factor 2 | | Factor 3 | | Factor 4 | | Uniqueness | |
| MATRICS Sustained Attention |  | 0.886 |  | -0.088 |  | 0.275 |  | -0.073 |  | 0.396 |  |
| MATRICS Working Memory |  | 0.782 |  | 0.004 |  | 0.058 |  | -0.008 |  | 0.434 |  |
| MATRICS Processing Speed |  | 0.738 |  | 0.055 |  | -0.217 |  | 0.146 |  | 0.249 |  |
| MATRICS Verbal Learning |  | 0.562 |  | 0.037 |  | -0.157 |  | 0.043 |  | 0.570 |  |
| MATRICS Problem Solving |  | 0.510 |  | -0.115 |  | -0.158 |  | 0.038 |  | 0.605 |  |
| MATRICS Visual Learning |  | 0.225 |  | 0.146 |  | -0.475 |  | -0.041 |  | 0.620 |  |
| PANSS WALLWORK DISORGANIZED |  | -0.136 |  | 0.029 |  | 0.469 |  | 0.291 |  | 0.538 |  |
| PANSS WALLWORK EXCITED |  | 0.083 |  | 0.158 |  | -0.029 |  | 0.481 |  | 0.720 |  |
| PANSS WALLWORK NEGATIVE |  | 0.079 |  | 0.271 |  | 0.650 |  | -0.183 |  | 0.469 |  |
| PANSS WALLWORK DEPRESSED |  | -0.077 |  | 0.838 |  | 0.005 |  | 0.028 |  | 0.275 |  |
| MADRS Depression |  | 0.041 |  | 0.812 |  | 0.318 |  | -0.034 |  | 0.106 |  |
| ROSENBERG Self-Esteem |  | 0.020 |  | -0.617 |  | 0.058 |  | -0.040 |  | 0.628 |  |
| YMRS Mania |  | -0.014 |  | -0.099 |  | -0.179 |  | 0.840 |  | 0.326 |  |
| PANSS WALLWORK POSITIVE |  | -0.014 |  | -0.051 |  | 0.232 |  | 0.544 |  | 0.617 |  |
|  | | | | | | | | | | | |
| Note. Applied rotation method is promax. | | | | | | | | | | | |

Table S5: Cross-level correlations of the hierarchical factor analysis.

| Source | Target | Correlation |
| --- | --- | --- |
| Cognition 1 | Cognition 2 | 0.971177 |
| Cognition 1 | Depression/Negative 2 | -0.485368 |
| Cognition 2 | Cognition 3 | 0.997764 |
| Cognition 2 | Depression/Negative 3 | -0.369863 |
| Cognition 2 | Thought Disorder 3 | -0.164420 |
| Depression/Negative 2 | Cognition 3 | -0.235840 |
| Depression/Negative 2 | Depression/Negative 3 | 0.988760 |
| Depression/Negative 2 | Thought Disorder 3 | 0.385103 |
| Cognition 3 | Cognition 4 | 0.979692 |
| Cognition 3 | Depression/Negative 4 | -0.121221 |
| Cognition 3 | Detachment 4 | -0.779663 |
| Cognition 3 | Thought Disorder 4 | -0.150374 |
| Depression/Negative 3 | Cognition 4 | -0.191995 |
| Depression/Negative 3 | Depression/Negative 4 | 0.959022 |
| Depression/Negative 3 | Detachment 4 | 0.643687 |
| Depression/Negative 3 | Thought Disorder 4 | 0.239691 |
| Thought Disorder 3 | Cognition 4 | -0.116926 |
| Thought Disorder 3 | Depression/Negative 4 | 0.282320 |
| Thought Disorder 3 | Detachment 4 | 0.192432 |
| Thought Disorder 3 | Thought Disorder 4 | 0.997050 |

Note. The number after the factor name identifies the level in the hierarchy.
